# Medical Student Screening of Social Determinants of Health in Urogynecology Surgical Patients

**DOI:** 10.31486/toj.25.0096

**Published:** 2026

**Authors:** Isabel K. Anderson, Erin Biggs, Meenakshi Mishra, Hannah H. Jordan, Leise R. Knoepp

**Affiliations:** ^1^ The University of Queensland Medical School, Ochsner Clinical School, New Orleans, LA; ^2^ Center for Outcomes and Health Services Research, Ochsner Clinic Foundation, New Orleans, LA; ^3^ Department of Urogynecology, Ochsner Clinic Foundation, New Orleans, LA

**Keywords:** *Education–medical–undergraduate*, *physician engagement*, *preventive medicine*, *risk assessment*, *risk factors*, *social determinants of health*, *socioeconomic factors*

## Abstract

**Background:**

Growing evidence documents the impacts that social determinants of health (SDoH) can have on medical and surgical outcomes, but many physicians report lacking the time to screen and address patients’ SDoH. This gap creates an opportunity for medical students to apply their knowledge of SDoH in a clinical setting, actively participate as members of the medical team, and address a critical aspect of patient care.

**Methods:**

In a pilot project, medical students rotating on the urogynecology surgical service were tasked with calling patients the day prior to their surgical encounter and asking them SDoH screening questions. To evaluate the completion of SDoH screening by medical students, a data analysis team determined if medical students added or updated patients’ SDoH data in the medical record within 72 hours preoperatively or by 24 hours postoperatively.

**Results:**

Between May 21 and October 31, 2024, 18 medical students screened 44 patients for social risk factors. Excluding tobacco use and transportation needs screening, medical students conducted at least 90% of the SDoH screenings for these patients.

**Conclusion:**

This project demonstrates that medical students are a resource that can be used to increase the rates of patient SDoH screening, particularly for factors that are not routinely screened. In addition, this project demonstrates how SDoH screening can be efficiently implemented on a busy surgical service without interrupting patient flow.

## INTRODUCTION

Since the establishment of the Commission on Social Determinants of Health by the World Health Organization in 2005,^1^ the importance of social determinants of health (SDoH)—the nonmedical factors that influence health outcomes—has become widely recognized in the health care sector.^2^ SDoH such as education status and income level can drive up to 80% of health outcomes, with research showing that the number of deaths attributable to social factors in the United States is comparable to the number of deaths attributed to pathophysiological and behavioral causes.^3,4^ While SDoH are often viewed as being principally relevant to primary care and preventive medicine, growing evidence indicates that SDoH can have a measurable impact on surgical outcomes.^5^ For example, Bennett et al found that patients with the lowest socioeconomic status had the greatest operative mortality, with a single-level increase in socioeconomic status correlating to a 7.1% mean decrease in operative mortality risk.^6^ Wright et al reported that lower levels of health literacy were associated with longer hospital lengths of stay for patients who underwent abdominal surgery.^7^

To address adverse SDoH (ie, social risk factors), surgical patients must first be screened, but relatively little focus has been placed on screening surgical patients.^8^ Physicians have reported lacking the time to screen patients for SDoH and connect them with resources.^9^ This gap creates an opportunity for medical students to apply their knowledge of SDoH in a clinical setting and actively participate as members of the medical team. A 2020 study showed that medical students who conducted SDoH screening of surgical patients reported increased self-perceived value, confidence in interacting with patients and assessing their SDoH, and positive perceptions of surgery as a career.^10^

In 2020, Ochsner Health launched the Healthy State initiative, a collaborative effort among health care, community, education, and policy organizations, to address the leading causes of poor health in Louisiana.^11^ A key goal of the Healthy State initiative is to raise awareness of the social drivers of health. In response, The University of Queensland-Ochsner Clinical School chapter of the Gold Humanism Honor Society began an educational initiative in 2021 focused on increasing third- and fourth-year medical students’ awareness of SDoH and health equity via a peer-led orientation presentation. However, this presentation did not cover how to log SDoH data within the electronic health record (EHR) system nor did it address any potential barriers to patient SDoH screening faced by medical students. Further, this initiative demonstrated a need for medical student screening of SDoH to be incorporated into provider workflows and to be encouraged, if not expected, by attending physicians with whom medical students rotate.

As a result, a pilot project was initiated in partnership with an attending provider on the urogynecology surgical service. The provider incorporated medical student screening of patients’ SDoH into the workflow and provided education on how to use the SDoH screening tool in the EHR system.

## METHODS

The Ochsner Health Institutional Review Board (IRB) reviewed this study and determined that it did not involve human subject research.

### Project Description

Ochsner Health uses the Epic (Epic Systems Corporation) EHR system. In 2022, Epic, in partnership with the White House, integrated a standardized SDoH screening tool into the EHR system that includes questions related to 12 key SDoH: tobacco use, financial resource strain, transportation needs, stress, depression, health literacy, alcohol use, food insecurity, physical activity, housing stability, utilities, and social isolation.^12^ Searching “SDoH” in a patient record will return the Social Determinants of Health Spotlight. Providers can then select the arrow next to each SDoH variable to access the related screening questions.

Beginning in May 2024, third-year and elective fourth-year medical students assigned to rotate with the participating attending physician on the urogynecology surgical service were tasked with calling surgical patients the day prior to their surgical encounter (or on Friday if the surgical encounter was scheduled for Monday) and asking them the SDoH screening questions. If the medical student was unable to contact the patient the day prior to the surgical encounter, the student was instructed to ask the questions during the preoperative phase, or if necessary, during the postoperative hospitalization period. On the day of surgery, the medical student and surgical team members identified social risk factors and discussed management options for the patient's health-related social needs. Management options included referrals to social work, tobacco cessation counseling, and community resource connections (eg, food pantries, Alcoholics Anonymous). This workflow is depicted in the Figure.

**Figure.**
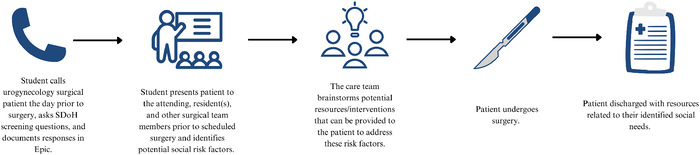
Steps show the process of incorporating social determinants of health (SDoH) screening into the urogynecology surgery workflow.

Medical students were provided with instructions on how to access the SDoH screening questions via a document posted on the Blackboard Ob-Gyn course website (Appendix). Students rotating with the participating attending physician were required to participate in this project. Student expectations were communicated via the Blackboard Ob-Gyn course website and by the Ob-Gyn resident assigned to the urogynecology service. Students were directed to ask all the screening questions for the 12 SDoH to each of the surgical patients on the participating attending physician's service for the days on which they were assigned to rotate.

### Evaluation Plan

To evaluate the completion of SDoH screening by medical students, statisticians and data analysts from the Ochsner Center for Outcomes and Health Services Research (the data analysis team) determined if urogynecology surgical patients’ SDoH data were added or updated by medical students within 72 hours preoperatively and 24 hours postoperatively. The data analysis team identified surgical patients from the urogynecology surgery schedule and identified medical students by their identified role within the Epic system. The evaluation period was defined as May 21 to October 31, 2024 (ie, from the start of the project to the end of medical student rotations for the semester).

### Outcomes

Three outcomes were measured in this project (Table 1). The first outcome was the percentage of total surgical cases in which an SDoH element was recorded by any medical professional (ie, any individual involved in the health care process, including medical students). The second outcome was the percentage of total surgical cases in which medical students specifically recorded an SDoH element. The results of these 2 outcomes were used to calculate the third outcome, the proportion of SDoH recorded by medical students.

**Table 1.** Project Outcomes and Method of Calculation

Outcome	Calculation
Percentage of total surgical cases in which an SDoH element was recorded by any medical professional^a^	**Numerator**: Number of SDoH recorded by any medical professional^a^ **Denominator**: Total surgical cases during the period May 21 to October 31, 2024^b^
Percentage of total surgical cases in which an SDoH element was recorded by medical students	**Numerator**: Number of SDoH recorded by medical students **Denominator**: Total surgical cases during the period May 21 to October 31, 2024^b^
Proportion of SDoH recorded by medical students	**Numerator**: Number of SDoH recorded by medical students **Denominator**: Number of SDoH recorded by any medical professional^a^

^a^Any medical professional means any individual involved in the health care process, including medical students.

^b^The total number of surgical cases during the period May 21 to October 31, 2024, was 104.

SDoH, social determinants of health.

The data analysis team collected 4 key variables to assess these outcomes: (1) a unique patient identification number generated by Epic (for privacy purposes and IRB compliance, this number was not the same as patient's medical record number); (2) the patient's surgery date; (3) the patient's SDoH screened by any medical professional within the 96-hour time period; (4) the patient's SDoH screened by a medical student within the 96-hour time period.

## RESULTS

Between May 21 and October 31, 2024, 104 surgical cases were completed on the urogynecology service. Forty-four of these patients were screened for social risk factors by 18 medical students. Among the 104 patients, 76 were screened for tobacco use by any medical professional, with medical students accounting for 23 of those screens. Thus, the proportion of tobacco use screenings conducted by medical students was 30.3%. Forty-eight patients were asked the transportation needs screening questions, with medical students responsible for 43 of the screenings, thus making the medical student screening proportion 89.6%. For financial resource strain, health literacy, and utilities, 48 patients were screened, of whom 44 were screened by medical students, accounting for 91.7% of all screenings. For both stress and depression, medical students accounted for 96.7% of screenings (29 of 30 patients). Medical students were responsible for 95.5% of screenings for alcohol use (42 of 44 patients) and 95.6% of screenings for physical activity (43 of 45 patients). For food insecurity and housing stability, medical students conducted 91.5% of screenings (43 of 47 patients). Finally, of the 45 patients screened for social isolation, 43 were screened by medical students (95.6%). Thus, for all SDoH except tobacco use and transportation needs, medical students were responsible for more than 90% of patient screenings. These findings are summarized in Table 2.

**Table 2.** Social Determinants of Health Screening of Patients Undergoing Urogynecology Surgery

Social Determinant of Health	SDoH Recorded by Any Medical Professional^a^, n	% of Total Surgical Cases^b^	SDoH Recorded by Medical Students, n	% of Total Surgical Cases^b^	Proportion of SDoH Recorded by Medical Students,^c^ %
Tobacco use	76	73.1	23	22.1	30.3
Financial resource strain	48	46.2	44	42.3	91.7
Transportation needs	48	46.2	43	41.3	89.6
Stress	30	28.8	29	27.9	96.7
Depression	30	28.8	29	27.9	96.7
Health literacy	48	46.2	44	42.3	91.7
Alcohol use	44	42.3	42	40.4	95.5
Food insecurity	47	45.2	43	41.3	91.5
Physical activity	45	43.3	43	41.3	95.6
Housing stability	47	45.2	43	41.3	91.5
Utilities	48	46.2	44	42.3	91.7
Social isolation	45	43.3	43	41.3	95.6
Any SDoH	81	77.9	44	42.3	54.3

^a^Any medical professional means any individual involved in the health care process, including medical students.

^b^The total number of surgical cases during the period May 21 to October 31, 2024, was 104.

^c^Number of SDoH recorded by medical students / Number of SDoH recorded by any medical professional.

SDoH, social determinants of health.

## DISCUSSION

This project demonstrates that medical students are a resource that can be used to increase the rates of patient SDoH screening, especially for determinants that are not routinely screened by other health care providers, such as stress, depression, and social isolation. This project also demonstrates how SDoH screening can be efficiently implemented on a busy surgical service without interrupting patient flow.

However, this project also identified potential areas for improvement in the documentation and tracking of SDoH for data analysis.

We found that if a patient provides the same response to an SDoH screening question at 2 or more discrete time points, Epic will only include the initial response and will not show that the patient was screened again for that social determinant. This reporting anomaly in Epic may explain the difference between the total percentage of surgical patients screened for tobacco use overall (73.1%) vs the total percentage screened by medical students (22.1%). Another potential explanation is that only some of the SDoH screening questions—none related to tobacco use—are displayed when a user clicks the arrow symbol next to the heading Social Determinants of Health in the Epic SDoH Spotlight (see step 4 in the Appendix).

In addition, data collection did not consider whether a medical student was scheduled on the urogynecology service for each of the 104 surgical procedures, which may explain why only 44 surgical patients were screened by medical students. An additional limitation of this project is that because of scheduling, not every third-year medical student is assigned to rotate on the urogynecology service. For those who are assigned to rotate on urogynecology, the rotation duration is generally only 1 week per student. Fourth-year elective students may rotate on the service for a longer period (usually 2 to 4 weeks), but the majority of students who rotate on the service are third-year students. Thus, most medical students were unable to follow up at postoperative visits on the social risk factors they had identified. Further, the structure of the rotation hindered the ability of students to identify and adequately address social risk factors at patients’ initial clinic visits prior to surgery, as the majority of students met the patients undergoing surgery on the scheduled procedure date.

Another key issue with implementing this project was documentation of SDoH in Epic. Students must type “SDoH” in the search bar at the top of a patient's chart to access the Social Determinants of Health Spotlight. Having the tool visible upon opening the chart by including it in the Snapshot tab or Med Student tab would address the access concern and serve as a reminder to screen patients. As noted previously, clicking the arrow next to Social Determinants of Health in the Spotlight does not pull up all the screening questions and omits questions relating to depression and tobacco use. Thus, users must navigate back to the Social Determinants of Health Spotlight after completing one set of screening questions and click the arrows next to each determinant to access the related question sets. This issue could be resolved by including all screening questions in a pop-up box that appears after clicking the arrow next to Social Determinants of Health. Despite updates to the Ochsner Health Epic EHR system since the implementation of this project, these issues have persisted.

One of the data analysis challenges in this project was that SDoH data are not stored in the same location in Epic. Data related to tobacco use and alcohol use are stored in history, while data for the other drivers are in flowsheets. Adjusting the storage of SDoH data so that all determinants are in the same location would improve data analysis efficiency and allow additional projects that involve the use of patient SDOH data to be conducted.

## CONCLUSION

This project demonstrated the feasibility of tasking medical students with SDoH screening and incorporating the screening process into a specific service line workflow. This project can be expanded by working with providers across disciplines to develop additional workflows that incorporate SDoH screening by medical students. For example, medical students on surgical and hospital medicine rotations could screen patients when they are pre-rounding, develop plans to address identified social needs, and present those plans during rounds. A potential research opportunity is analyzing patient outcomes subsequent to medical students’ screening and addressing social risk factors. Research could also be conducted to assess student experiences with this project.

## Notes

*This article meets the Accreditation Council for Graduate Medical Education and the American Board of Medical Specialties Maintenance of Certification competencies for Patient Care and Medical Knowledge.*

## Appendix. Instructions Provided to Medical Students on How to Access Social Determinants of Health Screening Questions in the Electronic Medical Record

*Note: All images in this appendix are from Epic Playground and are not from an actual patient record.*

1. The day before scheduled surgeries, call your patients (from an Ochsner phone line) to ask them the SDoH questions.
  - **For surgeries scheduled on Monday, call on Friday afternoon.**
  - To find the patient's phone number, hover over the patient's name.
2. To find the SDoH questions, type “SDoH” in the search bar inside the patient's chart and click the search icon.
3. This search will pull up the Social Determinants of Health Spotlight.
4. Click the arrow symbol next to EACH social determinant. (Note: Clicking the arrow symbol next to the heading Social Determinants of Health won’t pull up all the questions.) Start with Tobacco Use and once finished asking the tobacco-related questions, search “SDOH” again and continue down the list.
5. Next to each question is a paper icon (circled in red) where you can write comments.
6. The attending will ask you to identify each patient's SDoH, and you will help brainstorm potential interventions that can be provided to address the patient's social needs.
7. Based on identified risk factors, you will be assigned to enter information in the patient's discharge instructions. Go to the discharge tab and click the Patient Inst tab (outlined in red) on the left.
8. To insert information, click the image icon (circled in red).
9. This action will pull up a dialog box. Click Import (outlined in red).
10. Import images related to the SDoH identified for the patient. To find these images, access the shared OneDrive. Download the relevant images and save them to your desktop.
